# 2,2′-(Biphenyl-2,2′-diyldi­oxy)diaceto­hydrazide

**DOI:** 10.1107/S1600536808014864

**Published:** 2008-05-21

**Authors:** Farooq Ibad, Asra Mustafa, Muhammad Raza Shah, Donald VanDerveer

**Affiliations:** a HEJ Research Institute of Chemistry, International Center for Chemical and Biological Sciences, University of Karachi, Karachi 75270, Pakistan; b Chemistry Department, Clemson University, Clemson, SC 29634-0973, USA

## Abstract

In the mol­ecule of the title compound, C_16_H_18_N_4_O_4_, the dihedral angle between the mean planes of the two benzene rings is 56.76 (5)°. The crystal structure reveals extensive inter­molecular hydrogen bonds between carbonyl O atoms and primary amines, as well as between primary and secondary amines of hydrazide, forming rings of *R*_2_^2^(10) and *R*_2_^2^(6) motifs, respectively. The structure is further stabilized by intra­molecular and non-classical hydrogen bonds of the types N—H⋯O and C—H⋯O, respectively. The structure does not show any π–π inter­actions.

## Related literature

For related literature see: Dekeyser *et al.* (2003 ▶); Ali *et al.* (2008 ▶); Baudry *et al.* (2006 ▶); Bhat *et al.* (1974 ▶); Etter (1990 ▶); Kakefuda *et al.* (2002 ▶); Litvinchuk *et al.* (2004 ▶); Priebe *et al.* (2008 ▶); Sisson *et al.* (2006 ▶); Thaker & Patel (2008 ▶); Yan *et al.* (1993 ▶).
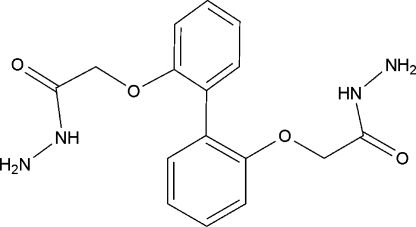


## Experimental

### 

#### Crystal data

C_16_H_18_N_4_O_4_
*M* *_r_* = 330.34
Triclinic, 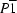

*a* = 8.4041 (17) Å
*b* = 9.7148 (19) Å
*c* = 10.465 (2) Å
α = 99.27 (3)°
β = 92.50 (3)°
γ = 113.85 (3)°
*V* = 765.7 (3) Å^3^
*Z* = 2
Mo *K*α radiation
μ = 0.10 mm^−1^
*T* = 153 (2) K
0.31 × 0.29 × 0.22 mm

#### Data collection

Rigaku Mercury CCD diffractometer
Absorption correction: multi-scan (REQAB; Jacobson, 1998 ▶) *T*_min_ = 0.968, *T*_max_ = 0.977
5673 measured reflections
2695 independent reflections
2440 reflections with *I* > 2σ(*I*)
*R*_int_ = 0.009

#### Refinement

*R*[*F* ^2^ > 2σ(*F* ^2^)] = 0.036
*wR*(*F* ^2^) = 0.092
*S* = 1.05
2695 reflections
237 parameters
6 restraints
H atoms treated by a mixture of independent and constrained refinement
Δρ_max_ = 0.20 e Å^−3^
Δρ_min_ = −0.20 e Å^−3^

### 

Data collection: *CrystalClear* (Rigaku/MSC, 2006 ▶); cell refinement: *CrystalClear*; data reduction: *CrystalClear*; program(s) used to solve structure: *SHELXS97* (Sheldrick, 2008 ▶); program(s) used to refine structure: *SHELXTL* (Sheldrick, 2008 ▶); molecular graphics: *SHELXTL*; software used to prepare material for publication: *SHELXTL*.

## Supplementary Material

Crystal structure: contains datablocks I, global. DOI: 10.1107/S1600536808014864/pv2081sup1.cif

Structure factors: contains datablocks I. DOI: 10.1107/S1600536808014864/pv2081Isup2.hkl

Additional supplementary materials:  crystallographic information; 3D view; checkCIF report

## Figures and Tables

**Table 1:** Hydrogen-bond geometry (Å, °)

*D*—H⋯*A*	*D*—H	H⋯*A*	*D*⋯*A*	*D*—H⋯*A*
N1—H1*A*⋯O4^i^	0.924 (14)	2.192 (15)	3.059 (2)	155.7 (15)
N1—H1*B*⋯O4^ii^	0.930 (14)	2.510 (17)	3.0112 (18)	114.1 (13)
N3—H3⋯N4^iii^	0.900 (13)	2.191 (15)	2.9524 (18)	141.9 (14)
N4—H4*B*⋯O1^iv^	0.936 (14)	2.267 (16)	2.9873 (18)	133.3 (14)
N1—H1*B*⋯O1	0.930 (14)	2.348 (17)	2.7873 (18)	108.6 (13)
N2—H2⋯O2	0.906 (14)	2.118 (17)	2.5375 (16)	107.1 (13)
N3—H3⋯O3	0.900 (13)	2.230 (16)	2.5977 (17)	103.9 (12)
C5—H5*A*⋯N1^v^	0.95	2.53	3.359 (2)	146
C11—H11*A*⋯O1^vi^	0.95	2.47	3.292 (2)	145

Symmetry codes: (i) 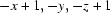; (ii) 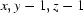; (iii) 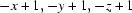; (iv) 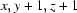; (v) 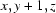; (vi) 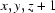.

**Fig. 1.**
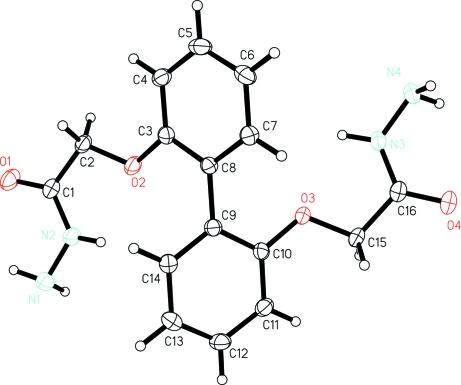
Molecular structure of the the title compound showing 50% probability displacement ellipsoids with arbitrary shperes for H atoms.

**Fig. 2.**
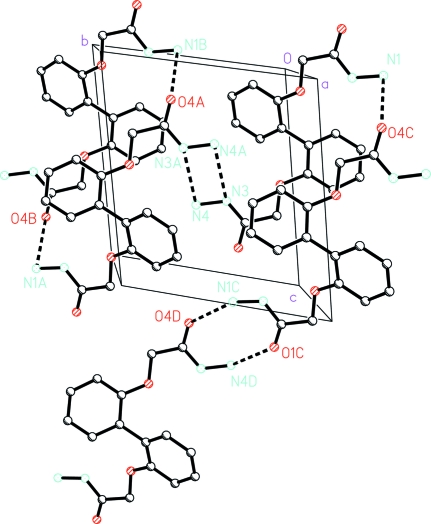
Packing diagram of (I) with hydrogen bonds viewed along the b axis. Symmetry codes for A through D are: 1 - x,1 - y,1 - z; x,1 + y,z; 1 - x,-y,1 - z; and 1 - x,1 - y,2 - z, respectively.

## supplementary crystallographic information

### Comment

Biphenyl hydrazides are of crucial importance in the design and synthesis of novel advanced functional materials (Thaker & Patel, 2008) and compounds of biological importance (Kakefuda *et al.*, 2002; Dekeyser *et al.*, 2003). Our interest in the synthesis of biphenyl dihydrazide arose from the fact that we wanted to attach macrocycles like porphyrin to diphenyl dicarboxylic acid and carboxylic substituted oligo(*p*-phenylene)s (Litvinchuk *et al.*, 2004) to form functionalized pores (Sisson *et al.*, 2006; Baudry *et al.*, 2006). The coupling of amino-substituted macrocycles gave poor yields so we changed the strategy and synthesized carboxylic substituted macrocycles and hydrazide substituted biphenyls. Studies on the coupling of these biphenyl hydrazides and macrocycles are in progress. In this paper, we report the synthesis and crystal structure of the title compound, (I).

The molecules of the title compound (Fig. 1) are held together by intermolecular hydrogen bonds involving carbonyl O-atoms and primary amines as well as primary amines and secondary amines of the type N—H···O and N—H···H, respectively, which stabilize the crystal structure (Fig. 2) resulting in ten and six membered which may be described in the graph set notation as *R*_2_^2^(10) and *R*_2_^2^(6) (Etter, 1990). There are three intramolecular hydrogen bonds in addition to non-classical hydrogen bonds involving phenyl H-atoms and a carbonyl oxygen and a primary amine; details of hydrogen bonding geometry have been provided in Table 1.

The C1—O1 and C16—O4 distances in (I) are 1.2284 (17)Å and 1.2338 (16) Å, respectively, typical of double bonds (Yan *et al.*, 1993), whereas the distances C1—N2 and C16—N3 at 1.3320 (18)Å and 1.3291 (18)Å are consistent with those reported (Priebe *et al.*, 2008), suggesting partial double bond character. Similarly, the distances N1—N2 and N3—N4, 1.4198 (17)Å and 1.4196 (17) Å, respectively, are typical for a single bond, which are in agreement with those of the analogous compound (Bhat *et al.*, 1974), suggesting that the title compound exists as resonance hybrid between a polar and a neutral form.

### Experimental

Diethyl 2,2'-(biphenyl-2,2'-diylbis(oxy))diacetate (500 mg, 1.4 mmol) was refluxed in the presence of hydrazine hydrate (5 ml, 103 mmol) in ethanol (10 ml) at 353 K for 2 h, the reaction mixture was cooled down to room temperature and then poured into 10 ml of water. The reaction mixture was extracted three times with ethyl acetate. The combined organic phases were concentrated under reduced pressure. The crude residue was dissolved in ethanol and slow evaporation of ethanol afforded colorless crystals (276 mg, 60% yield) suitable for XRD analysis.

### Refinement

Positions of the amine H atoms were located from difference Fourier maps and were allowed to refine with *U*_iso_(H) = 1.2*U*_eq_ (parent N-atom). The remaining H atoms were geometrically placed and treated as riding atoms with C—H = 0.95 Å (aryl) and 0.98 Å (methylene), and *U*_iso_(H) = 1.2*U*_eq_ (parent C-atom).

### Crystal data

**Table:**

C_16_H_18_N_4_O_4_	*Z* = 2
*M**_r_* = 330.34	*F*_000_ = 348
Triclinic, *P*1	*D*_x_ = 1.433 Mg m^−^^3^
Hall symbol: -P 1	Mo *K*α radiation λ = 0.71073 Å
*a* = 8.4041 (17) Å	Cell parameters from 2518 reflections
*b* = 9.7148 (19) Å	θ = 2.7–26.4º
*c* = 10.465 (2) Å	µ = 0.11 mm^−^^1^
α = 99.27 (3)º	*T* = 153 (2) K
β = 92.50 (3)º	Chip, colorless
γ = 113.85 (3)º	0.31 × 0.29 × 0.22 mm
*V* = 765.7 (3) Å^3^	

### Data collection

**Table:**

Rigaku Mercury CCD diffractometer	2695 independent reflections
Radiation source: Sealed Tube	2440 reflections with *I* > 2σ(*I*)
Monochromator: Graphite Monochromator	*R*_int_ = 0.009
Detector resolution: 14.6306 pixels mm^-1^	θ_max_ = 25.2º
*T* = 153(2) K	θ_min_ = 2.0º
ω scans	*h* = −10→9
Absorption correction: multi-scan(REQAB; Jacobson, 1998)	*k* = −10→11
*T*_min_ = 0.968, *T*_max_ = 0.977	*l* = −12→12
5673 measured reflections	

### Refinement

**Table:**

Refinement on *F*^2^	Secondary atom site location: difference Fourier map
Least-squares matrix: full	Hydrogen site location: inferred from neighbouring sites
*R*[*F*^2^ > 2σ(*F*^2^)] = 0.036	H atoms treated by a mixture of independent and constrained refinement
*wR*(*F*^2^) = 0.092	*w* = 1/[σ^2^(*F*_o_^2^) + (0.0447*P*)^2^ + 0.3477*P*] where *P* = (*F*_o_^2^ + 2*F*_c_^2^)/3
*S* = 1.05	(Δ/σ)_max_ = 0.001
2695 reflections	Δρ_max_ = 0.20 e Å^−^^3^
237 parameters	Δρ_min_ = −0.20 e Å^−^^3^
6 restraints	Extinction correction: none
Primary atom site location: structure-invariant direct methods	

### Special details

**Table:**

Geometry. All e.s.d.'s (except the e.s.d. in the dihedral angle between two l.s. planes) are estimated using the full covariance matrix. The cell e.s.d.'s are taken into account individually in the estimation of e.s.d.'s in distances, angles and torsion angles; correlations between e.s.d.'s in cell parameters are only used when they are defined by crystal symmetry. An approximate (isotropic) treatment of cell e.s.d.'s is used for estimating e.s.d.'s involving l.s. planes.
Refinement. Refinement of *F*^2^ against ALL reflections. The weighted *R*-factor *wR* and goodness of fit *S* are based on *F*^2^, conventional *R*-factors *R* are based on *F*, with *F* set to zero for negative *F*^2^. The threshold expression of *F*^2^ > σ(*F*^2^) is used only for calculating *R*-factors(gt) *etc*. and is not relevant to the choice of reflections for refinement. *R*-factors based on *F*^2^ are statistically about twice as large as those based on *F*, and *R*- factors based on ALL data will be even larger.

### Fractional atomic coordinates and isotropic or equivalent isotropic displacement parameters (Å^2^)

**Table:**

	*x*	*y*	*z*	*U*_iso_*/*U*_eq_	
O1	0.32268 (15)	−0.23766 (13)	−0.20890 (9)	0.0317 (3)	
O2	0.26996 (14)	−0.02884 (11)	0.08294 (9)	0.0263 (2)	
O3	0.33765 (13)	0.10884 (10)	0.50896 (8)	0.0242 (2)	
O4	0.41183 (15)	0.35080 (12)	0.81794 (9)	0.0313 (3)	
N1	0.28627 (18)	−0.42946 (14)	−0.03033 (12)	0.0282 (3)	
H1A	0.389 (2)	−0.419 (2)	0.0137 (16)	0.034*	
H1B	0.293 (2)	−0.452 (2)	−0.1191 (14)	0.034*	
N2	0.28707 (16)	−0.28115 (14)	−0.00437 (11)	0.0253 (3)	
H2	0.253 (2)	−0.254 (2)	0.0732 (15)	0.035 (5)*	
N3	0.41999 (16)	0.39435 (13)	0.61151 (11)	0.0236 (3)	
H3	0.428 (2)	0.362 (2)	0.5272 (14)	0.038 (5)*	
N4	0.46353 (18)	0.55390 (14)	0.64554 (12)	0.0271 (3)	
H4A	0.544 (2)	0.5916 (19)	0.7182 (15)	0.032*	
H4B	0.364 (2)	0.565 (2)	0.6732 (16)	0.032*	
C1	0.29940 (17)	−0.19832 (16)	−0.09628 (12)	0.0216 (3)	
C2	0.28191 (18)	−0.04884 (16)	−0.05358 (12)	0.0217 (3)	
H2B	0.3851	0.0379	−0.0726	0.026*	
H2C	0.1756	−0.0523	−0.1013	0.026*	
C3	0.21044 (17)	0.07599 (15)	0.14010 (12)	0.0196 (3)	
C4	0.20643 (18)	0.19491 (15)	0.08338 (13)	0.0224 (3)	
H4C	0.2437	0.2059	−0.0002	0.027*	
C5	0.14735 (18)	0.29724 (16)	0.15019 (14)	0.0253 (3)	
H5A	0.1430	0.3779	0.1116	0.030*	
C6	0.09474 (18)	0.28244 (15)	0.27275 (14)	0.0246 (3)	
H6A	0.0544	0.3527	0.3181	0.029*	
C7	0.10118 (17)	0.16440 (15)	0.32919 (13)	0.0212 (3)	
H7A	0.0664	0.1557	0.4137	0.025*	
C8	0.15766 (16)	0.05875 (14)	0.26424 (12)	0.0187 (3)	
C9	0.15926 (17)	−0.07024 (15)	0.32413 (12)	0.0187 (3)	
C10	0.25033 (17)	−0.04279 (15)	0.44785 (12)	0.0189 (3)	
C11	0.25260 (18)	−0.16331 (16)	0.50320 (13)	0.0229 (3)	
H11A	0.3164	−0.1431	0.5864	0.028*	
C12	0.1608 (2)	−0.31304 (16)	0.43565 (14)	0.0261 (3)	
H12A	0.1631	−0.3956	0.4724	0.031*	
C13	0.0658 (2)	−0.34330 (16)	0.31497 (14)	0.0271 (3)	
H13A	0.0014	−0.4462	0.2700	0.033*	
C14	0.06546 (18)	−0.22235 (15)	0.26023 (13)	0.0229 (3)	
H14A	0.0000	−0.2436	0.1776	0.028*	
C15	0.36921 (19)	0.14095 (16)	0.64783 (12)	0.0242 (3)	
H15A	0.4720	0.1228	0.6757	0.029*	
H15B	0.2663	0.0717	0.6837	0.029*	
C16	0.40281 (17)	0.30626 (16)	0.69949 (12)	0.0218 (3)	

### Atomic displacement parameters (Å^2^)

**Table:**

	*U*^11^	*U*^22^	*U*^33^	*U*^12^	*U*^13^	*U*^23^
O1	0.0463 (7)	0.0413 (6)	0.0184 (5)	0.0285 (5)	0.0086 (4)	0.0069 (4)
O2	0.0425 (6)	0.0299 (5)	0.0148 (5)	0.0231 (5)	0.0048 (4)	0.0048 (4)
O3	0.0358 (6)	0.0189 (5)	0.0135 (4)	0.0073 (4)	0.0008 (4)	0.0026 (4)
O4	0.0438 (6)	0.0325 (6)	0.0156 (5)	0.0157 (5)	0.0017 (4)	0.0004 (4)
N1	0.0377 (7)	0.0248 (6)	0.0259 (6)	0.0165 (6)	0.0055 (5)	0.0054 (5)
N2	0.0358 (7)	0.0251 (6)	0.0193 (6)	0.0164 (5)	0.0066 (5)	0.0051 (5)
N3	0.0307 (6)	0.0196 (6)	0.0179 (6)	0.0087 (5)	0.0049 (5)	0.0008 (4)
N4	0.0358 (7)	0.0210 (6)	0.0224 (6)	0.0111 (5)	0.0055 (5)	0.0004 (5)
C1	0.0205 (6)	0.0282 (7)	0.0173 (6)	0.0112 (6)	0.0020 (5)	0.0045 (5)
C2	0.0250 (7)	0.0252 (7)	0.0150 (6)	0.0103 (6)	0.0024 (5)	0.0051 (5)
C3	0.0210 (6)	0.0182 (6)	0.0184 (6)	0.0079 (5)	−0.0007 (5)	0.0016 (5)
C4	0.0239 (7)	0.0216 (7)	0.0204 (6)	0.0071 (5)	0.0010 (5)	0.0075 (5)
C5	0.0266 (7)	0.0184 (7)	0.0311 (7)	0.0081 (6)	−0.0006 (6)	0.0094 (6)
C6	0.0265 (7)	0.0183 (7)	0.0300 (7)	0.0116 (6)	0.0013 (6)	0.0026 (5)
C7	0.0215 (7)	0.0202 (7)	0.0208 (6)	0.0080 (5)	0.0014 (5)	0.0032 (5)
C8	0.0197 (6)	0.0159 (6)	0.0185 (6)	0.0058 (5)	−0.0006 (5)	0.0029 (5)
C9	0.0217 (6)	0.0187 (7)	0.0179 (6)	0.0101 (5)	0.0051 (5)	0.0044 (5)
C10	0.0222 (6)	0.0173 (6)	0.0169 (6)	0.0080 (5)	0.0051 (5)	0.0025 (5)
C11	0.0281 (7)	0.0259 (7)	0.0183 (6)	0.0138 (6)	0.0036 (5)	0.0071 (5)
C12	0.0351 (8)	0.0219 (7)	0.0271 (7)	0.0154 (6)	0.0063 (6)	0.0102 (6)
C13	0.0337 (8)	0.0175 (7)	0.0289 (7)	0.0102 (6)	0.0016 (6)	0.0030 (5)
C14	0.0279 (7)	0.0207 (7)	0.0200 (6)	0.0105 (6)	0.0003 (5)	0.0028 (5)
C15	0.0325 (7)	0.0252 (7)	0.0134 (6)	0.0104 (6)	0.0021 (5)	0.0041 (5)
C16	0.0204 (6)	0.0251 (7)	0.0176 (6)	0.0080 (5)	0.0017 (5)	0.0019 (5)

### Geometric parameters (Å, °)

**Table:**

O1—C1	1.2284 (17)	C4—H4C	0.9500
O2—C3	1.3736 (16)	C5—C6	1.385 (2)
O2—C2	1.4242 (15)	C5—H5A	0.9500
O3—C10	1.3779 (17)	C6—C7	1.3901 (19)
O3—C15	1.4259 (15)	C6—H6A	0.9500
O4—C16	1.2338 (16)	C7—C8	1.3922 (19)
N1—N2	1.4198 (17)	C7—H7A	0.9500
N1—H1A	0.925 (14)	C8—C9	1.4931 (18)
N1—H1B	0.930 (14)	C9—C14	1.3949 (19)
N2—C1	1.3320 (18)	C9—C10	1.4037 (19)
N2—H2	0.907 (14)	C10—C11	1.3937 (19)
N3—C16	1.3291 (18)	C11—C12	1.387 (2)
N3—N4	1.4196 (17)	C11—H11A	0.9500
N3—H3	0.904 (14)	C12—C13	1.386 (2)
N4—H4A	0.914 (14)	C12—H12A	0.9500
N4—H4B	0.936 (14)	C13—C14	1.3888 (19)
C1—C2	1.5164 (19)	C13—H13A	0.9500
C2—H2B	0.9900	C14—H14A	0.9500
C2—H2C	0.9900	C15—C16	1.514 (2)
C3—C4	1.3924 (19)	C15—H15A	0.9900
C3—C8	1.4046 (18)	C15—H15B	0.9900
C4—C5	1.389 (2)		
			
C3—O2—C2	119.52 (10)	C7—C6—H6A	120.1
C10—O3—C15	117.33 (10)	C6—C7—C8	121.29 (12)
N2—N1—H1A	105.6 (11)	C6—C7—H7A	119.4
N2—N1—H1B	106.5 (11)	C8—C7—H7A	119.4
H1A—N1—H1B	107.7 (15)	C7—C8—C3	117.98 (12)
C1—N2—N1	122.76 (12)	C7—C8—C9	120.86 (11)
C1—N2—H2	119.5 (11)	C3—C8—C9	121.16 (12)
N1—N2—H2	116.3 (11)	C14—C9—C10	117.93 (12)
C16—N3—N4	123.00 (11)	C14—C9—C8	120.81 (11)
C16—N3—H3	121.4 (12)	C10—C9—C8	121.23 (12)
N4—N3—H3	114.3 (12)	O3—C10—C11	122.79 (11)
N3—N4—H4A	106.8 (11)	O3—C10—C9	116.07 (11)
N3—N4—H4B	107.6 (11)	C11—C10—C9	121.13 (12)
H4A—N4—H4B	105.1 (15)	C12—C11—C10	119.35 (12)
O1—C1—N2	123.62 (13)	C12—C11—H11A	120.3
O1—C1—C2	120.88 (12)	C10—C11—H11A	120.3
N2—C1—C2	115.50 (11)	C13—C12—C11	120.59 (13)
O2—C2—C1	108.04 (11)	C13—C12—H12A	119.7
O2—C2—H2B	110.1	C11—C12—H12A	119.7
C1—C2—H2B	110.1	C12—C13—C14	119.61 (13)
O2—C2—H2C	110.1	C12—C13—H13A	120.2
C1—C2—H2C	110.1	C14—C13—H13A	120.2
H2B—C2—H2C	108.4	C13—C14—C9	121.34 (12)
O2—C3—C4	123.65 (12)	C13—C14—H14A	119.3
O2—C3—C8	115.15 (11)	C9—C14—H14A	119.3
C4—C3—C8	121.17 (12)	O3—C15—C16	109.67 (11)
C5—C4—C3	119.38 (13)	O3—C15—H15A	109.7
C5—C4—H4C	120.3	C16—C15—H15A	109.7
C3—C4—H4C	120.3	O3—C15—H15B	109.7
C6—C5—C4	120.42 (12)	C16—C15—H15B	109.7
C6—C5—H5A	119.8	H15A—C15—H15B	108.2
C4—C5—H5A	119.8	O4—C16—N3	124.25 (13)
C5—C6—C7	119.76 (13)	O4—C16—C15	119.25 (12)
C5—C6—H6A	120.1	N3—C16—C15	116.49 (11)
			
N1—N2—C1—O1	−5.0 (2)	C7—C8—C9—C10	−55.87 (18)
N1—N2—C1—C2	174.76 (12)	C3—C8—C9—C10	125.09 (14)
C3—O2—C2—C1	−163.98 (11)	C15—O3—C10—C11	−25.80 (18)
O1—C1—C2—O2	−175.55 (12)	C15—O3—C10—C9	155.02 (12)
N2—C1—C2—O2	4.71 (16)	C14—C9—C10—O3	−178.44 (11)
C2—O2—C3—C4	−18.76 (19)	C8—C9—C10—O3	−0.38 (18)
C2—O2—C3—C8	163.23 (12)	C14—C9—C10—C11	2.38 (19)
O2—C3—C4—C5	−178.61 (12)	C8—C9—C10—C11	−179.57 (12)
C8—C3—C4—C5	−0.7 (2)	O3—C10—C11—C12	179.79 (12)
C3—C4—C5—C6	0.7 (2)	C9—C10—C11—C12	−1.1 (2)
C4—C5—C6—C7	0.0 (2)	C10—C11—C12—C13	−0.8 (2)
C5—C6—C7—C8	−0.8 (2)	C11—C12—C13—C14	1.2 (2)
C6—C7—C8—C3	0.82 (19)	C12—C13—C14—C9	0.1 (2)
C6—C7—C8—C9	−178.24 (12)	C10—C9—C14—C13	−1.9 (2)
O2—C3—C8—C7	178.01 (11)	C8—C9—C14—C13	−179.96 (13)
C4—C3—C8—C7	−0.05 (19)	C10—O3—C15—C16	−159.34 (11)
O2—C3—C8—C9	−2.93 (18)	N4—N3—C16—O4	4.7 (2)
C4—C3—C8—C9	179.01 (12)	N4—N3—C16—C15	−175.62 (12)
C7—C8—C9—C14	122.12 (14)	O3—C15—C16—O4	171.27 (12)
C3—C8—C9—C14	−56.91 (18)	O3—C15—C16—N3	−8.42 (17)

### Hydrogen-bond geometry (Å, °)

**Table:**

*D*—H···*A*	*D*—H	H···*A*	*D*···*A*	*D*—H···*A*
N1—H1A···O4^i^	0.924 (14)	2.192 (15)	3.059 (2)	155.7 (15)
N1—H1B···O4^ii^	0.930 (14)	2.510 (17)	3.0112 (18)	114.1 (13)
N3—H3···N4^iii^	0.900 (13)	2.191 (15)	2.9524 (18)	141.9 (14)
N4—H4B···O1^iv^	0.936 (14)	2.267 (16)	2.9873 (18)	133.3 (14)
N1—H1B···O1	0.930 (14)	2.348 (17)	2.7873 (18)	108.6 (13)
N2—H2···O2	0.906 (14)	2.118 (17)	2.5375 (16)	107.1 (13)
N3—H3···O3	0.900 (13)	2.230 (16)	2.5977 (17)	103.9 (12)
C5—H5A···N1^v^	0.95	2.53	3.359 (2)	146
C11—H11A···O1^vi^	0.95	2.47	3.292 (2)	145

Symmetry codes: (i) −*x*+1, −*y*, −*z*+1; (ii) *x*, *y*−1, *z*−1; (iii) −*x*+1, −*y*+1, −*z*+1; (iv) *x*, *y*+1, *z*+1; (v) *x*, *y*+1, *z*; (vi) *x*, *y*, *z*+1.
